# Primordial Media: The Shrouded Realm of Composite Materials

**DOI:** 10.1021/acsphotonics.5c02910

**Published:** 2026-03-09

**Authors:** Viktor A. Podolskiy, Evgenii Narimanov

**Affiliations:** † Department of Physics and Applied Physics, 14710 University of Massachusetts Lowell, Lowell, Massachusetts 01854, United States; ‡ Department of ECE, 311308 Purdue University, West Lafayette, Indiana 47907, United States

**Keywords:** spatial dispersion, nonlocality, metamaterials, composites, effective medium theory, additional waves, nonlocal transfer matrix method

## Abstract

Electromagnetic composites (metamaterials) recently underwent explosive growth fueled in part by advances in nanofabrication. It is commonly believed that as the size of the components decreases, the behavior of a composite converges to the response of a homogeneous material (recent research indicates that in the limit of nanoscale composites, the constituent parameters of nanostructures may be quantitatively affected by nonlocal corrections). Here we show that this intuitive understanding of the electromagnetic response of composite media is fundamentally flawed, even at the qualitative level. In contrast to the well-understood (local) effective medium response, the properties of nanostructured composites can be dominated, not simply corrected, by electromagnetic nonlocality. We demonstrate that in composites, the interplay between the nonlocality and the structural inhomogeneity introduces two fundamentally new electromagnetic regimes: primordial metamaterials and homogenizable nonlocality. We develop an analytical description of these regimes and show that the behavior of metamaterials in the limits of vanishing nonlocality and of vanishing component size does not commute. Our work opens a new dimension in the design space of nanostructured electromagnetic composites.

## Graphical abstract

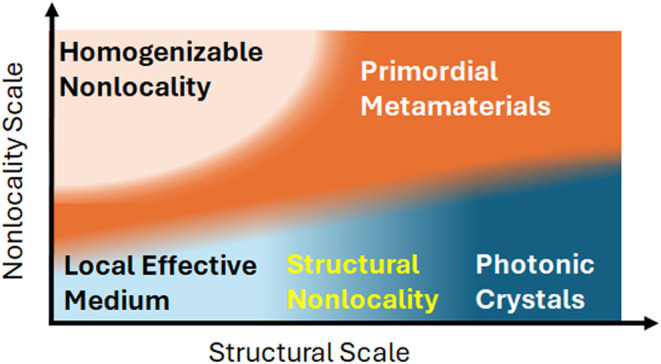

## Introduction

Composites with engineered optical response enable novel approaches to imaging, sensing, communications, and quantum engineering.^1–6^ In order to minimize the artifacts related to light interference and scattering on an individual component and to make the composite overall resemble homogeneous media,^7,8^ the feature size of recent composites approach few nanometers scale.^9–15^ Recent studies indicate, however, that the properties of nm-scale inclusions may deviate from their bulk response due to electromagnetic nonlocality.^16–24^ While these recent studies explored the regimes where few plasmonic particles are separated by nanometer-scale spacers, the broad design space where (local) permittivity and nonlocality of macroscopic materials are modulated in space remains largely unexplored. Here we demonstrate that the novel interactions between nonlocal components of the composite yield qualitative, not just quantitative change. Our analytical results, illustrated on the example of planar plasmonic metamaterials, while directly applicable to phononic, excitonic, and other nonlocal media, demonstrate the existence of two fundamentally new electromagnetic regimes in composites, primordial metamaterials, and homogenizable nonlocality.

The universal design space of electromagnetic composites that emerges from our work is illustrated in Figure 1. The bottom part of the figure contains the regimes that have been extensively studied over past decades^3–6,25–34^ and where inherent nonlocality is not important, photonic crystals, and local effective medium. The transition between these regimes can be described in terms of structural nonlocality that appears as a geometric correction to effective medium response of the inherently local composite. The upper part of the figure contains primordial metamaterials, a regime where the composite is dominated by the interference of inherently nonlocal additional waves and that has been recently realized in experiments.^35^ The final part of the design space is the homogenizable nonlocality, which has been unexplored up until now. The two new regimes, enabled by electromagnetic nonlocality, offer new opportunities in classical and quantum electrodynamics.

**Figure 1:**
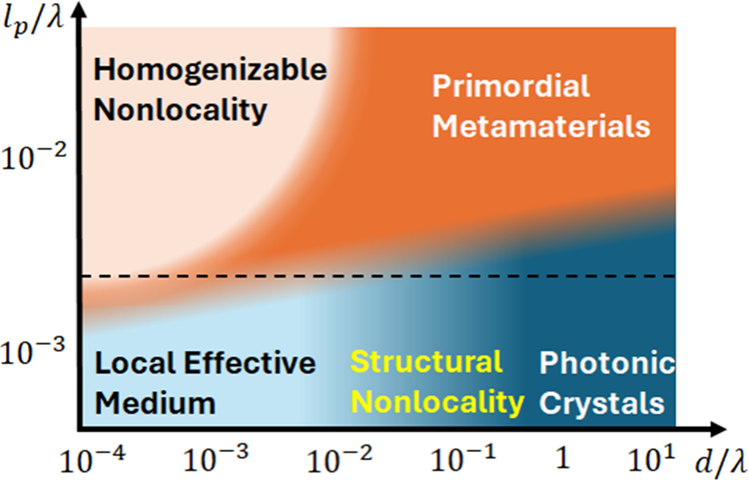
Interplay between the space inhomogeneity (parametrized by the composite length scale *d*, normalized to the vacuum wavelength λ) and the nonlocality (parametrized by the primordial length scale *l*_p_ normalized to λ) results in the complex optical response of the composites. When the nonlocality is sufficiently weak and can be ignored, the composites operate either in photonic crystal regime, dominated by interference of partially reflected light, or in *local effective medium* regime where the system behaves as homogeneous material. The transition from the photonic crystal to local effective medium can be described in terms of structural nonlocality^8,26,27,36–38^ where the nonlocal part of effective permittivity tensor results from geometric correction of the response of inherently local composite; this regime is outside the scope of this work. Increasing nonlocality drives the composite into a *primordial metamaterial* regime that is dominated by the interference of additional electromagnetic waves. Very fine-scaled strongly nonlocal composites yield *homogenizable nonlocality* regime where composite behaves as a homogeneous, but strongly nonlocal-media. The dashed line illustrates the parameter range explored in this work.

## Results and Discussion

### Electromagnetic Response of Nonlocal Composites

Electromagnetic nonlocality can be attributed to the inherent motion of charges within materials,^39–41^ necessarily imposed in any media by the fundamental quantum uncertainty principle.^39,42^ This phenomenon, previously analyzed in macroscopic homogeneous materials^43,44^ and in individual nanostructures^18,19^ can be described by introducing the dependence of the dielectric permittivity on the wavevector *k⃗* in addition to its dependence on operating frequency ω.^39^ This dependence raises the degree of dispersion relations describing plane waves propagating in materials, making them at least ∝*k* ^4^ instead of ∝*k* ^2^ (see Appendix for details) and thereby introducing additional electromagnetic waves. The existence of these waves reflects the fundamental difference between the inherently nonlocal response of actual materials and their simplified description in terms of local (frequency-dependent) permittivity and permeability.

In contrast to previous studies that mostly focused on a single homogeneous nonlocal layer or a single nonlocal nanostructure,^19–22,41,43,44^ here we consider light propagation through a nonlocal composite material with spatially dependent nonlocality. Although the new regimes of nonlocal composites presented in this work reflect the general and universal properties of nanostructured media, to better illustrate our results, we use an example of nonmagnetic (*B⃗* = *H⃗*) weakly nonlocal material that is homogeneous in the *xy* plane and is inhomogeneous and nonlocal in the *z* direction; the formalism presented in this work can be extended to other geometries of inhomogeneity or nonlocality.

Since describing nonlocality in the wavevector domain is known to lead to complications when materials’ properties depend on position,^40,45^ we use the representation of the nonlocal behavior in the spatial domain, where materials’ response is expanded in derivatives of the electric field. For materials with inversion symmetry, the lowest-possible nonlocality appears as the second derivative of the electric field.^40^ The operator of electric energy of the material, defined as $w_{e}=\frac{1}{16\pi }\int \mathrm{E⃗}\cdot \mathrm{D⃗}d^{3}r=\frac{1}{16\pi }\int \mathrm{E⃗}\cdot \mathrm{\epsilon ̂}\mathrm{E⃗}d^{3}r$ , has to be a self-adjoined operator. Therefore, the real space dependence of electric displacement on the electric field takes the form:

1 $$\mathrm{D⃗}=\mathrm{\epsilon ̂}\mathrm{E⃗}-\frac{c^{2}}{\omega^{2}}\frac{\partial }{\partial z}\left(\alpha \left(z\right)\frac{\partial E_{z}}{\partial z}\right)ẑ$$

with ϵ̂ being local part of material response. For illustration purposes, we limit the discussion below to uniaxial materials with optical axes along the *ẑ* direction. Therefore, tensor ϵ̂ is diagonal with nonzero components ϵ_*xx*_ = ϵ_*yy*_ = ϵ_⊥_, and ϵ_*zz*_. The dimensionless parameter α describes the nonlocality of the electromagnetic response of the material.

At the fundamental level, this inherent nonlocality is related to the motion of the free carriers or to the fundamental quantum mechanical position uncertainty of the localized charges. The charge displacement over one period of optical excitation introduces a characteristic spatial scale *l*_p_ = λ*v*/*c* where λ and *c* are wavelength and speed of light in vacuum, respectively, and *v* is characteristic speed of charges in the material. In dielectric media whose response is driven by bound electrons, *l*_p_ is typically small, below a few nanometers, and is determined by electron delocalization scale in chemical bonds. A variety of well-known and recently discovered materials support extended charge excitations that result in strongly nonlocal response. For example, in plasmonic media, the charge motion is dominated by Fermi velocity *v*_*f*_, resulting in *l*_p_ ∼ 10···100 nm^35,42^ (see below). In excitonic materials, $v∼\sqrt{\hbar \omega_{o}/m^{*}}$ ^46^ with ω_0_ and m* being the excitonic frequency and effective mass of the exciton, respectively, with wide range of *l*_p_ accessible depending on the origin of excitonic or polaritonic response.^47–52^ Notably, *l*_p_ ∼ 1 μm have been reported in phononic materials.^43^

The ratio of the corresponding spatial scale *l*_p_ to the free space wavelength defines α = *l*_*p*_^2^/λ^2^. This single parameter is capable of adequately describing the nonlocal response of materials, independent of the origin of the underlying nonlocality.

Since in our geometry the material is homogeneous and isotropic in the *xy* plane, we look for propagating waves that have harmonic dependence [*E⃗*, *D⃗*··· ∝ exp­(−*i*ω*t* + *ik*_*x*_ *x*)], thereby fixing *xz* plane as the propagation plane, and focusing on propagation of transverse-magnetic (TM-polarized) modes that have *B⃗*∥*ŷ* as these are the only waves that are affected by anisotropy and nonlocality in our geometry (see Appendix).

Starting from Maxwell equations in Cartesian coordinates

2 $$\begin{array}{l} \frac{\partial H_{y}}{\partial z}=i\frac{\omega }{c}\epsilon_{⊥}E_{x},\\ \frac{\partial E_{x}}{\partial z}-ik_{x}E_{z}=i\frac{\omega }{c}B_{y},\\ ik_{x}H_{y}=-i\frac{\omega }{c}D_{z}, \end{array}$$

and eliminating the electric field, we obtain the following equation governing the *B*_*y*_ component

3 $$\left(\epsilon_{\mathrm{zz}}-\frac{c^{2}}{\omega^{2}}\frac{\partial }{\partial z}\alpha \left(z\right)\frac{\partial }{\partial z}\right)\left[\frac{\partial }{\partial z}\left\{\frac{1}{\epsilon_{⊥}}\frac{\partial B_{y}}{\partial z}\right\}+\frac{\omega^{2}}{c^{2}}B_{y}\right]-k_{x}^{2}B_{y}=0$$

This equation represents one of the main results of the present work. In materials with a smooth variation of the permittivity and nonlocality, eq 3 can be used to calculate the position-dependent distribution of the magnetic field across the composite, with the distributions of other field components given by eq 2. In particular, in homogeneous materials, eq 3 accepts plane wave solution with the resulting dispersion being identical to predictions of the wavenumber-dependent permittivity formalism (see Appendix).

For materials with step-continuous distributions of the permittivity, the requirement for eq 3 to have well-defined solutions [the requirement to have differentiable functions within eq 3] solves the decades-long puzzle of additional boundary conditions (ABCs)^40,41,45^ that are required to calculate the amplitudes of additional waves refracting through the interface.

The terms inside the curvy brackets of eq 3 require continuity of *B*_*y*_, and $\frac{1}{\epsilon_{⊥}}\frac{\partial B_{y}}{\partial z}$ , which as seen from eq 2 are equivalent to continuity of *D*_*z*_ and *E*_*x*_, respectively. These conditions are equivalent to boundary conditions that are imposed in the conventional (local) electromagnetism.^39^

It can be shown that the expression in the square brackets in eq 3 is proportional to *E*_*z*_. Therefore, both *E*_*z*_ and $\alpha \frac{\partial E_{z}}{\partial z}$ have to be also continuous through the interface of two nonlocal media. These two conditions represent the ABCs that are derived here purely from macroscopic electromagnetic description, without relying on quantum-mechanical description of materials.^40,41,43,46,53^ Further analysis reveals that when α → 0 on one side of the interface, the former of the two ABCs becomes redundant.

Importantly, the set of conventional and additional boundary conditions, derived in this work, ensure continuity of the normal component of Poynting flux through the interface, defined as^39^

4 Sz=c8πRe(ExHy*)−c2α16πω(Ez*∂Ez∂z−Ez∂Ez*∂z)

### Effective Medium Behavior of Composites

The eq 3 can be used to derive the response of composites in the effective medium limit, when the scale of variation of material parameters *d* → 0 and where the (averaged over the unit cell) fields are described by

5 $$\left(\varepsilon_{\mathrm{zz}}-\mathrm{\alpha ̃}\frac{c^{2}}{\omega^{2}}\frac{\partial^{2}}{\partial z^{2}}\right)\left[\frac{1}{\varepsilon_{⊥}}\frac{\partial^{2}B_{y}}{\partial z^{2}}+\frac{\omega^{2}}{c^{2}}B_{y}\right]-k_{x}^{2}B_{y}=0$$

with ε̂ and α̃ being effective medium parameters.

Enforcing the relatively slow (as compared with *d*) variation of continuous field components, we arrive to following expressions for the components of the effective permittivity tensor ε̂ of local composites

6 $$\varepsilon_{⊥}^{l}=\left\langle \epsilon_{⊥}\right\rangle ,\varepsilon_{\mathrm{zz}}^{l}=\langle 1/\epsilon_{\mathrm{zz}}\rangle^{-1}$$

with <···> representing average over the unit cell. These results are identical to effective medium parameters derived for multilayered metamaterials in previous studies.^7,54,55^

However, a similar procedure applied to nonlocal composites yields

7 $$\varepsilon_{⊥}^{\mathrm{nl}}=\left\langle \epsilon_{⊥}\right\rangle ,\varepsilon_{\mathrm{zz}}^{\mathrm{nl}}=\left\langle \epsilon_{\mathrm{zz}}\right\rangle ,\mathrm{\alpha ̃}=\langle 1/\alpha \rangle^{-1}$$

Importantly, this nonlocal effective medium composite *does not* behave as its local counterpart with a nonlocal correction. This is one of the central results of our work. To distinguish this new material regime from the other behaviors described in this work, we term this regime *homogenizable nonlocality*, highlighting the need for the underlying nonlocal response of the components as well as the resulting effective medium behavior of the composite. To illustrate how unusual this result is, we note that in homogenizable nonlocality regime the local part of permittivity of a multilayer stack (known to be the foundational platform for extremely anisotropic—hyperbolic—metamaterials^4,52,55–57^ ) becomes isotropic, with anisotropy appearing only in nonlocal dielectric response of the composite.

The fact that ε̂^l^ ≠ ε̂^*nl*^ indicates that there must be a nontrivial transition region between the two effective medium regimes. Here we identify this region as the primordial metamaterials regime.

## The Breakdown of Local Electromagnetism in Composites

To gain insight into the dramatic effect of weak nonlocality on the optical response of composites, we calculate light transmission through a stack of increasingly thinner layers. While the behavior described below does not rely on the origin of nonlocal response and therefore will be manifested in a variety of polaritonic, plasmonic, and excitonic composites, here we consider a representative example of plasmonic/nonlocal dielectric layers, keeping the total thickness of the stack and the concentration of plasmonic layers constant, while decreasing the size of each individual layer and increasing the total number of layers.

We assume that the nonlocal response of plasmonic layers is well described by the permittivity of the degenerate Fermi gas^42^

8 $$\epsilon_{p}=1-\frac{\omega_{p}^{2}}{\omega \left(\omega +i\tau \right)}-\frac{3\omega_{p}^{4}v_{f}^{2}k_{z}^{2}}{5c^{2}\omega^{2}{\left(\omega +i\tau_{\mathrm{nl}}\right)}^{2}}$$

where ω_p_ is the plasma frequency, *v*_f_ is the Fermi velocity, and the parameters τ and τ_nl_ describe the scattering losses in local and nonlocal regimes,^42^ with *v*_*f*_^2^/*c* ^2^ ∼ 10^–5^, τ = 0.1ω_p_, τ_nl_ = 0.2ω_p_, representing typical behavior of noble metals^16,18,19,58^ and degenerately doped semiconductors^59,60^ in the vicinity of their respective plasma frequencies. The properties of the dielectric material components are assumed to be independent of wavelength, with ϵ_d_ = 2 + 10^–6^ (5 + 1*i*)*k*_*z*_^2^ *c* ^2^/ω^2^, representing a nonlocal analogue of dispersion-less transparent materials. Figure 2a illustrates the permittivity of the two materials used in this study.

**Figure 2:**
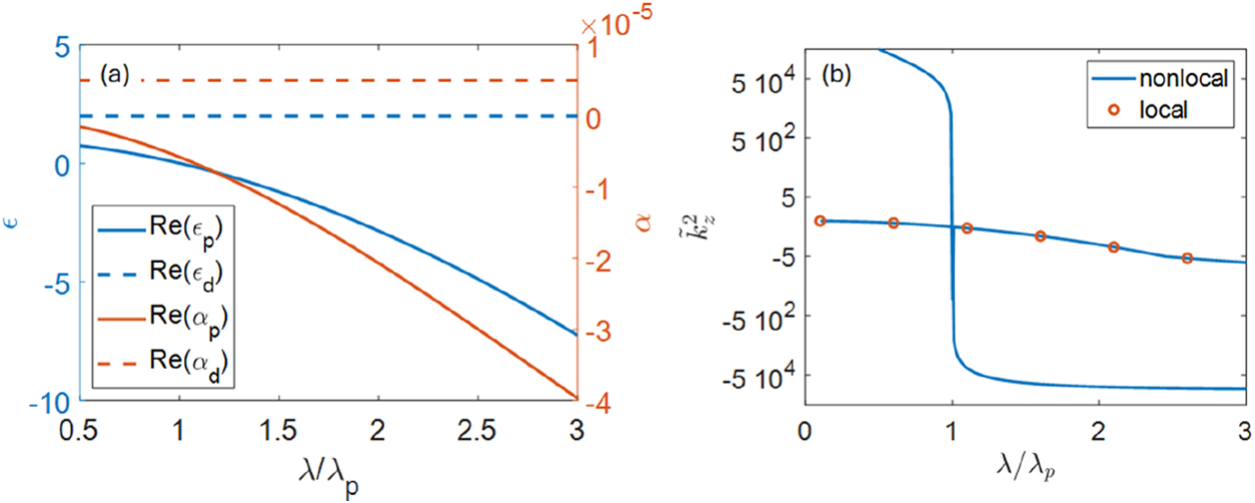
(a) Permittivity and nonlocality of plasmonic and dielectric layers used in this work; (b) (blue lines) dispersion of main and additional waves in plasmonic layers; red symbols illustrate dispersion of local plasmonic materials (α = 0).

In the remainder of the work, we normalize all linear dimensions by the plasma wavelength λ_p_ = 2π*c*/ω_p_ and describe the wavenumbers using the dimensionless wavevector *k̃*_β_ = *k*_β_ *c*/ω with β representing Cartesian components.

The dispersion of the main and additional waves in the plasmonic component of our composites is shown in Figure 2b. Note that both main and additional waves propagate for shorter wavelengths (*k̃*_*z*_^2^ > 0 for λ < λ_p_) while they exponentially decay for λ > λ_p_ (*k̃*_*z*_^2^ < 0). It is also seen that the dispersion of the main wave is well described by local permittivity model (α = 0) and that outside the epsilon-near-zero region, the additional wave has extremely large propagation constant, and as a result, it is not expected to couple to conventional diffraction-limited light. However, the wavenumber of the additional wave defines a new, primordial length scale *l*_p_ ∼ λ|*k̃*_*z*_| that, as we will show here, is crucial to understanding the response of nonlocal composites.

The coexistence of conventional waves and primordial excitations in the same material has a pronounced effect on its optical transmission and reflection spectra. Even when the sample thickness is well below the free-space wavelength, the interference of the propagating fields and the high-wavenumber primordial waves leads to pronounced oscillatory behavior in both reflection and transmission. In relatively thick nonlocal homogeneous media^43,44^ and in metamaterials with structural nonlocality^28,32,33,38^ such Fabry-Perot-like oscillations serve as a clear indication of nonlocal behavior. In composites with subwavelength thickness, these oscillations are the “smoking gun” for the excitation of primordial fields.

Specifically, Figure 3a illustrates the evolution of the transmission of TM-polarized light incident at 60° through a λ_p_/5-thick multilayer stack when nonlocality is ignored, and when the thickness of individual layers *d* changes from λ_p_/20 to λ_p_/10^4^. As expected, overall transmission through the composite does not depend on the (deeply subwavelength) thickness of its components, reflecting effective-medium-regime behavior; the dip at λ ≃ λ_p_ indicates the transition of the effective medium response from the elliptic *ε*_⊥_^*l*^, *ε*_*zz*_^*l*^ > 0 to the hyperbolic ε_⊥_^*l*^ > 0, ε_*zz*_^*l*^ < 0 regime.^55^

**Figure 3:**
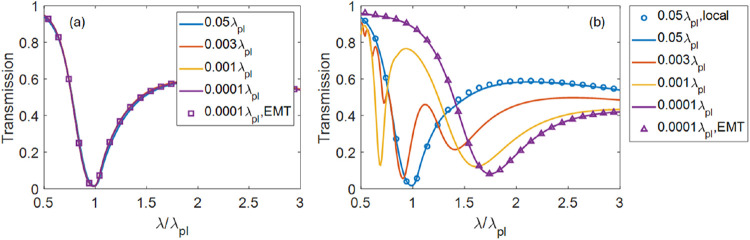
Transmission of TM-polarized light, incident at 60°, through λ_p_/5-thick stacks of plasmonic/dielectric multilayer composites, with different layer thicknesses *d*, calculated using (a) local and (b) nonlocal transfer matrix method, TMM [see Appendix]; squares and triangles represent results of local effective medium [eq 6] and homogenizable nonlocality [eq 7] description, respectively; circles in panel (b) correspond to the calculation using local TMM.

However, as seen in Figure 3b, this simple dynamics fundamentally changes when the nonlocality is taken into account. While for sufficiently large thickness (here, *d* ≳ 0.05λ_p_) the actual transmission closely follows the predictions of the local theory, for thinner layers the single transmission dip splits into multiple minima, one of which moves toward the longer wavelengths, while the other shifts in the opposite direction. Note that a similar oscillatory behavior has also been observed in the reflectance spectra of AlN/GaN heterostructures,^24^ and related to the nonlocality of the electromagnetic response of this polar material that arises from the hybridization of its LO phonons and surface phonon polaritons.^23^

Finally, when the layers reach *d* ∼ 10^–4^ λ_p_, the transmission spectrum once again converges to a spectrum featuring a single minimum. Here, the composite operates in homogenizable nonlocality regime.

### Evolution of Modes in Nonlocal Composites

To further understand the different regimes of the composites’ response, we analyze the modes propagating in periodically stratified bilayer composites. Dispersion of these modes can be analyzed by enforcing the Bloch periodicity relationship: *E⃗*(*z* + Δ) = *e* ^*ik̃*_*z*_Δ*ω*/*c*^ *E⃗*(*z*), *B⃗*(*z* + Δ) = *e* ^*ik̃*_*z*_Δ*ω*/*c*^ *B⃗*(*z*) with Δ being the period of the composite, resulting in the eigenvalue problem

9 $$\det\left|{\mathrm{T̂}}_{\Delta }-e^{i{\mathrm{k̃}}_{z}\Delta \omega /c}Î\right|=0$$

with *T̂*_Δ_ being the transfer matrix of one period of the composite (see Appendix).

Figure 4 illustrates the dispersion of the modes in relatively thick (*d* ≫ *l*_p_) composites calculated using local and nonlocal descriptions. It is clearly seen that local electromagnetism adequately describes propagation of the (main) mode, with the nonlocality only resulting in an additional wave that exponentially decays into the composite at a very small spatial scale.

**Figure 4:**
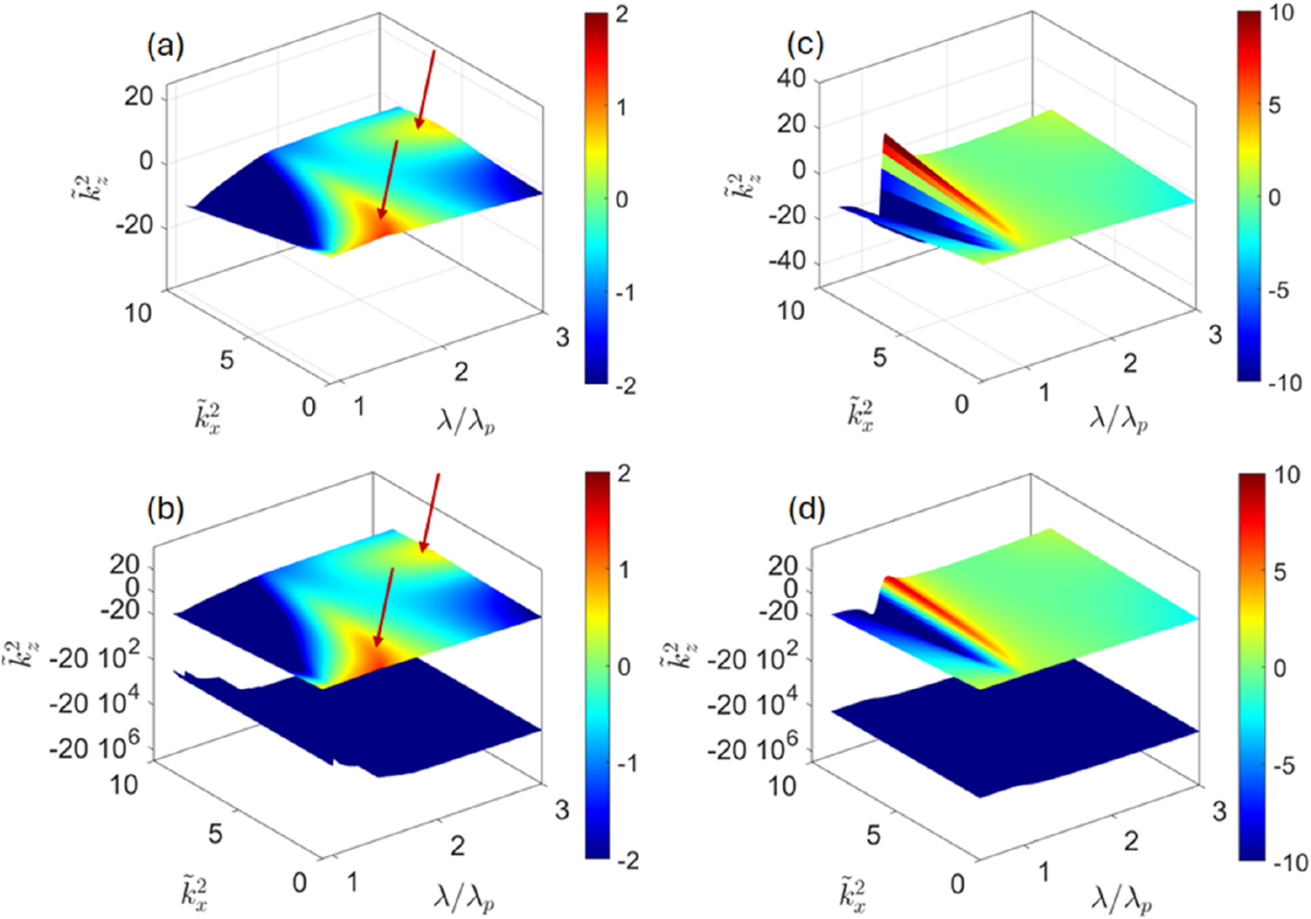
Dispersion of the modes in periodic plasmonic/dielectric composites operating in photonic crystal (a, b) and local effective medium theory (c, d) regimes. Layer thickness *d* = 0.3λ_p_ (a, b) and *d* = 0.05λ_p_ (c, d); λ/λ_p_, *k̃*_*x*_, and *k̃*_*z*_ represent dimensionless operating wavelength, in-plane and out-of-plane components of dimesionless wavenumber, respectively; *k̃*_*z*_^2^ > 0 corresponds to the propagating modes; *k̃*_*z*_^2^ < 0 represent exponentially decaying waves; panels (a, c) represent the results of local transfer matrix method and local effective medium theory, respectively; panels (b, d) represent the results of the nonlocal transfer matrix method. The two propagating modes seen in (a, b) (arrows) originate from the coupled surface plasmon polaritons;^61^ the sharp resonance in panels (c, d) corresponds to the transition between elliptic (λ < λ_p_) and hyperbolic (λ > λ_p_) regimes; see Supporting Information for a more detailed analysis of the optical properties of the composites in local effective medium regime.

The evolution of the material properties between *d* = 0.3λ_p_ and *d* = 0.05λ_p_ reflects the transition of the composite from a photonic crystal to local effective medium regimes. In the former regime, the behavior of composite is dominated by the interference of (partially) reflected light on the scale of the unit cell, resulting in a set of transparent (*k*_*z*_^2^ > 0) and “forbidden” (*k*_*z*_^2^ < 0) bands; the two propagating modes shown in Figure 4a,b corresponding to coupled surface plasmon polaritons propagating in metal-dielectric stacks.^8,61^

As the layer size is reduced below the (internal) wavelength, the response of the local composite converges to the predictions of the effective medium theory, eq 6, shown in Figure 4c,d. As mentioned above, and as described in refs 4,54,55,62 , here the multilayer composite behaves as a uniaxial material with elliptic (λ < λ_p_) or hyperbolic (λ > λ_p_) response. From this point on, further reduction of the layer thickness does not change the predictions of the local calculations.

However, when the layer thickness is decreased further and becomes comparable to the intrinsic component nonlocality scale *l*_p_, nonlocal calculations indicate fundamental changes in the composite behavior. This dynamics is illustrated in Figure 5. As the layer thickness is decreased, the additional wave initially moves closer to the main mode and begins to modulate its dispersion (Figure 5a). We note that while the changes in macroscopic response of composites due to underlying nonlocality of plasmonic structures reported here, are consistent with previous analysis with either quantum/hydrodynamic analysis^18,19,21,58,63,64^ or with a simplified approach presented in ref,^20^ these previous studies focused on the electromagnetism of either isolated plasmonic nanostructures or of a few-particle collections, separated by local spacers. As a result (see Discussion section), previous studies cannot be used to understand electromagnetism of extended materials with nonlocal components, the topic of this work.

**Figure 5:**
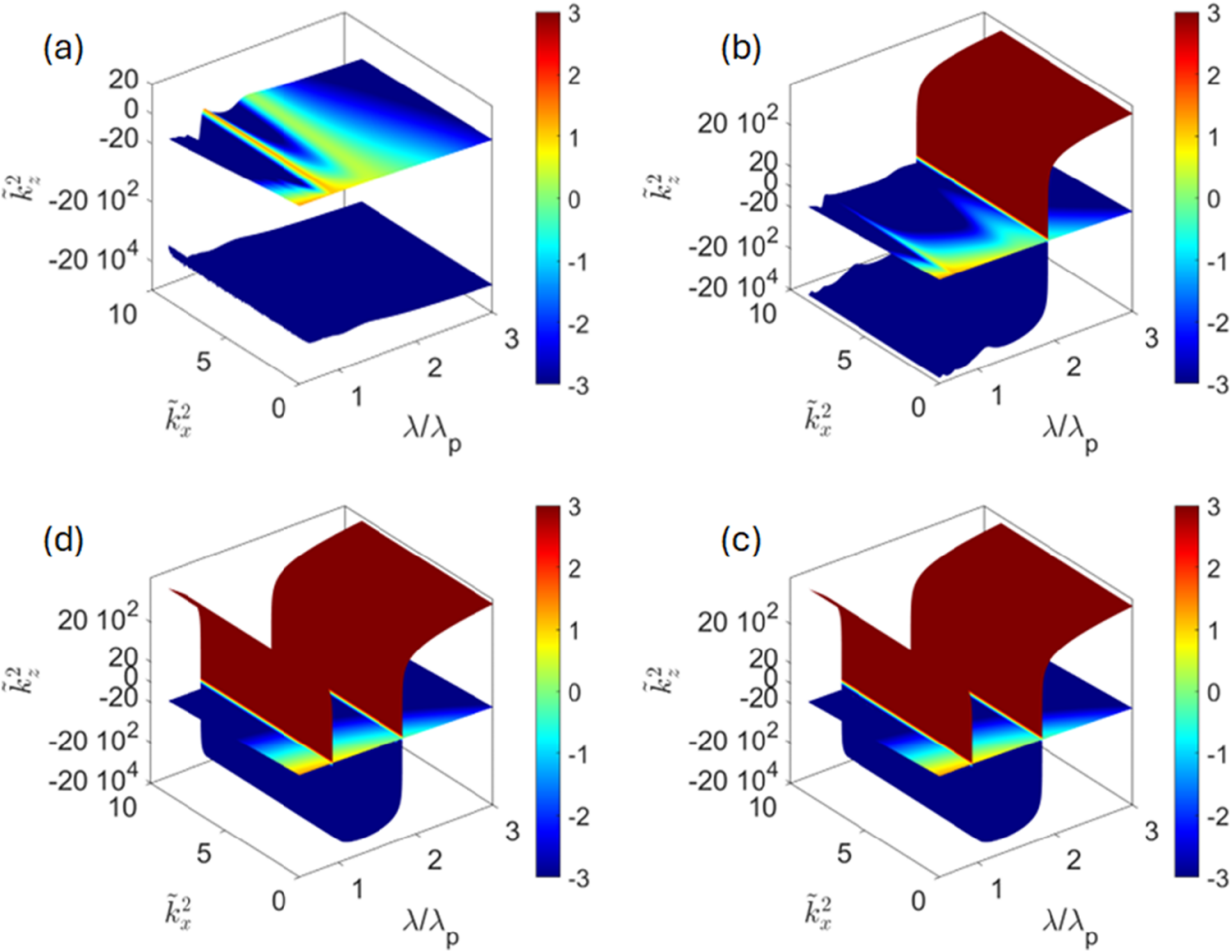
Dispersion of the modes in nonlocal plasmonic/dielectric composites operating in primordial metamaterial (a, b) and homogenizable nonlocality (c, d) regimes. Layer thickness *d* = 0.003λ_p_ (a), *d* = 10^–3^ λ_p_ (b), and *d* = 10^–4^ λ_p_ (c, d), calculated using nonlocal transfer matrix method (a–c) and homogenizable nonlocality (d); notice the difference from predictions of local effective medium theory (Figure 4c); see Supporting Information for a more detailed analysis of the optical properties of the composites in nonlocal regimes.

In thinner composites, the additional wave eventually changes its behavior from evanescent to propagating (Figure 5b and Supporting Information). The behavior of the composite across this regime, *primordial metamaterial*, originates from the interference of additional waves in metamaterial components, with the scale of the component being comparable to the wavelength of these additional waves. As a result, the optical response of primordial composites features highly oscillatory fields^35^ and is not described by effective medium theories. In a sense, the primordial metamaterial is a nonlocal analogue of the photonic crystal.

As the layer thickness is reduced further and becomes smaller than the nonlocality scale, the electromagnetic fields become smooth within the unit cell. In this regime (Figure 5c,d) the optical response of the composite converges to predictions of homogenizable nonlocality. The composite supports propagation of two plane-wave-like modes, whose dispersion is given by (eqs 5, 7). As seen from Figure 5d, homogenizable nonlocality perfectly describes propagation of waves in very finely structured nonlocal composites. By comparing Figure 5b to Figure 5c,d, it can be seen that the response of the composite in the long-wavelength limit homogenizes before its response in shorter wavelengths.

### Discussion

While nonlocal interactions have been a topic of extensive research for decades,^9,12,18,40,41,43,44,46,47,49,64^ the majority of previous works have either considered structural nonlocality that appears in fully local composites, or the response of isolated inherently nonlocal inclusions or of inclusions of separated by local spacers. Moreover, the majority of studies focusing on inherent nonlocality relied on material-specific nonlocality models. In contrast, here we present a unified, materials-agnostic framework for describing the electromagnetism of structurally inhomogeneous inherently nonlocal materials.

One of the important points of this work lies in the fact that the optical properties of composites are determined not simply by the relationship between the operating wavelength λ and the inhomogeneity scale *d* but by the complex interplay between λ, *d*, and the primordial nonlocality scale *l*_p_. The existence of *l*_p_ necessitates the primordial metamaterial regime and explains the fact that the limits of material response when *l*_p_ → 0 and *d* → 0 do not commute.

The composite operating in regime *l*_p_ ≪ *d* ≪ λ behaves according to the local effective medium theory; the composite operating in the regime *d* ≪ *l*_p_ ≪ λ behaves according to the rules of homogenizable nonlocality; the regime *d* ∼ *l*_p_ represents primordial metamaterial, dominated by the interference of additional waves and whose properties, as a result, are not described by an effective medium theory, even when the layers are very thin (see Table 1).

**Table 1:** Different Regimes of the Electromagnetic Response of Composites

	photonic crystal	local effective medium	primordial metamaterial	homogenizable nonlocality
length scales	*l*_p_ ≪ *d* ∼ λ	*l*_p_ ≪ *d* ≪ λ	*l*_p_ ∼ *d*	*d* ≪*l*_p_ ≪λ
continuous fields	*B*_*y*_, *E*_*x*_	*B*_*y*_, *E*_*x*_	*B*_*y*_, *E*_*x*_, $\alpha \frac{\partial E_{z}}{\partial z}$ , *E*_*z*_	*B*_*y*_, *E*_*x*_, $\alpha \frac{\partial E_{z}}{\partial z}$ , *E*_*z*_
homogenizable	no	yes, eq 6	no	yes, eq 7

The existence of the primordial metamaterial regime reconciles the different effective medium parameters predicted by eqs 6 and 7. Indeed, with *l*_p_ ≪ *d*, additional waves do not efficiently couple to the main modes. The local part of *D*_*z*_, ϵ_*zz*_ *E*_*z*_ slowly varies across the composite, resulting in eq 6.

In contrast, when *l*_p_ ≳ *d*, the displacement field is heavily influenced by nonlocal contributions and coupling between main and additional modes cannot be ignored. When *d* ≪ *l*_p_ ≪ λ, it is *E*_*z*_ continuity that dominates the homogenizable nonlocality response, resulting in eq 7. Similar to what is observed in local media,^34^ finite accuracy of the effective medium theories is expected to be increasingly more noticeable for thicker samples with nonlocal components.

As seen from eq 7, the effective nonlocality of the composite can be substantially different from the primordial nonlocality scale of its components, necessitating the introduction of two separate scales, *l*_p_ and $\lambda \sqrt{\mathrm{\alpha ̃}}$ , that describe the responses of components and of the composite overall, respectively. The material with weakest nonlocality defines the overall nonlocal scale of the composite, explaining why until very recently,^35^ deviations from local response at the room temperature have only been observed in single-nm environments. Indeed, both layers must be nonlocal to achieve primordial (or homogenizable) nonlocality responses in the bilayer metamaterials considered here. In the more complicated, multicomponent structures, the primordial response may manifest itself through cavity resonances of additional waves, surface analogs of additional waves, etc.; these phenomena may be realized without continuous nonlocal response throughout the composite.

Electromagnetism of primordial metamaterials is expected to strongly depend on the exact microstructure of the composite. Therefore, properties of aperiodic structures may strongly deviate from the properties of their periodic counterparts, similar to what has been observed in structurally nonlocal media.^32,33^

Lastly, it is important to note that while every material is nonlocal at its primordial scale, nonlocality in majority of dielectric materials is relatively weak (*l*_p_ is often of the order of nm at room temperature; the parameters used in this work yield *l*_p_ ∼ λ_p_/500, with *l*_p_ ∼ 20 nm for mid-infrared frequencies and *l*_p_ ∼ 2 nm for visible light). Therefore, the homogenizable nonlocality regime may be difficult to achieve in experiments since the bulk permittivity models are not applicable for single/few atomic-layer-structures. In contrast, primordial response should be widely available, especially in plasmonic, phononic, and excitonic materials that have *l*_p_ ≳ 10 nm. In composites based on highly nonlocal materials (where *l*_p_ ∼ λ),^43^ local effective medium regime may not be achievable with primordial response directly following local photonic crystal regime.

Table 1 describes not just the overall classification of material response but also the tools that can be used to analyze and optimize composites in a certain domain. For example, composites operating in primordial metamaterials regime must rely on numerically demanding brute-force analysis that resolves the internal structure of the composite and takes into account the nonlocality of each component. In contrast, local effective media or materials operating in homogenizable nonlocality regime can be analyzed with (often) much simpler descriptions that replace the composite with a homogeneous slab of material.

## Conclusions

To conclude, we have developed a theoretical formalism describing the electromagnetic response of composites having nonlocal components. We demonstrated that the design space for the electromagnetic response of such materials contains four fundamentally different regimes of optical behavior: local photonic crystals, local effective medium, primordial metamaterial, and homogenizable nonlocality. Finally, we explained the relationship between these regimes and the three length scales involved in the problem: layer size *d*, nonlocality scale *l*_p_, and wavelength scale λ.

Since nonlocality is an inherent property of any material, the two new regimes identified in this work, primordial metamaterial and homogenizable nonlocality, need to be considered in understanding and engineering of optical behavior of bulk nanostructured composites and laterally structured low-dimensional plasmonic and excitonic media.

Our results, illustrated here on (bicomponent) multilayered metamaterials with quadratic nonlocality, can be directly applied to more complicated layered media and can be generalized to different geometries (spherical, cylindrical, etc.) and to different nonlocality responses.

## Supplementary Material



## Notes

Analytical derivations of dispersion of waves in nonlocal materials as well as derivations of transfer- and scattering-matrix formalisms, and additional information about evolution of dispersion of the modes as the composite undergoes local to nonlocal transition (Appendix 1) (PDF)
