# Supplementary material for: Primordial Media: The Shrouded Realm of Composite Materials
Source: ACS Photonics. 2026 Mar 9;13(7):1910–8. doi: 10.1021/acsphotonics.5c02910 (PMC13047731; doi:10.1021/acsphotonics.5c02910)
Supplement: Supplementary file 1 [file ph5c02910_si_001.pdf]

Primordial Media: the shrouded realm of composite materials: Appendix

Viktor A Podolskiy[1] and Evgenii Narimanov[2]

[1]Department of Physics and Applied Physics, University of Massachusetts Lowell, Lowell, MA, 01854, USA
[2]Department of ECE, Purdue University, West Lafayette, IN, 47907 USA

February 9, 2026

5 pages
2 figures

Plane waves in nonlocal uniaxial materials

The dispersion of plane waves propagating through a homogeneous non-magnetic media can be found by substituting the plane-wave solution $[\vec{E}(\vec{r},t)=\vec{E}_0\exp(-i\omega t+i\vec{k}\cdot\vec{r})]$ into Maxwell equations and reducing these equations to the eigen value problem.[S1] When nonlocality is described in the wavenumber domain $[\hat{\epsilon}(\omega,\vec{k})]$, the above procedure yields:

$$\vec{k}(\vec{k}\cdot\vec{E}_0)-\vec{E}_0k^2+\hat{\epsilon}\frac{\omega^2}{c^2}\vec{E}_0=0. \tag{S1}$$

Here, the wavevector $\vec{k}$ and electric field magnitude $\vec{E}_0$ play the role of the eigenvalue and eigenvector, respectively.

For the case of uniaxial anisotropic media with weak spatial dispersion along its optical ($\hat{z}$) axis considered in this work, permittivity is described by a diagonal tensor $\hat{\epsilon}$ with non-vanishing components $\{\epsilon_\perp,\epsilon_\perp,\epsilon_{zz}+\alpha\tilde{k}_z^2\}$. As can be explicitly verified, Eq.(S1) yields two classes of solutions. The solutions of the first class have $\vec{E}_0\perp\hat{z}$. These solutions, also known as ordinary or transverse-electric (TE)-polarized waves are unaffected by anisotropy or nonlocality and have dispersion $\tilde{k}_x^2+\tilde{k}_y^2+\tilde{k}_z^2=\epsilon_\perp$.

The second class of solutions has $\vec{B}\perp\hat{z}$, and is therefore known as transverse-magnetic (TM)-polarized or extraordinary waves. The dispersion of these modes is given by:

$$\alpha\tilde{k}_z^4+(\epsilon_{zz}-\alpha\epsilon_\perp)\tilde{k}_z^2+\epsilon_\perp\left(\tilde{k}_x^2+\tilde{k}_y^2-\epsilon_{zz}\right)=0. \tag{S2}$$

Note that $\alpha\neq 0$ yields two TM-polarized modes with different dispersion (each of these modes can propagate in either $+\hat{z}$ or $-\hat{z}$ directions). In the limit of vanishingly small nonlocality $\alpha\to 0$, the dispersion of one of these modes (main wave) approaches the well-known dispersion of the extraordinary waves in homogeneous local uniaxial media $(\tilde{k}_x^2+\tilde{k}_y^2)/\epsilon_{zz}+\tilde{k}_z^2/\epsilon_\perp=1$, while the dispersion of the second (additional) wave diverges $\tilde{k}_z\to\infty$.

Transfer matrix method for nonlocal multilayered media

In optics, transfer matrix method (TMM)[S2] is often used to solve for light propagation in multilayered composites. Within this formalism (that can be extended to cylindrical, and other geometries), the solutions to Maxwell equations within each homogeneous layer are represented as a linear combination of (plane) waves, and boundary conditions are used to relate the amplitudes of these waves in neighboring layers via transfer matrices.

Over the years, multiple realizations of TMM[S3] have been developed. Here we utilize the TMM framework to analyze light propagation through composites that include nonlocal components. We follow the recipe of Ref.,[S2] and represent the fields in each layer as a linear combination of the [TM-polarized] waves propagating in $+\hat{z}$ and in $-\hat{z}$ directions with amplitudes $c^{\pm}_{l;1,2}$, with superscript defining the direction of the wave and subscripts defining the layer and the mode index (1=main wave, 2=additional wave), respectively. We assume that the permittivity of the layer l is given by the tensor with diagonal components by $\{\epsilon_{l,\perp},\epsilon_{l,\perp},\epsilon_{l,zz}+\alpha_lk_z^2c^2/\omega^2\}$ and that the interface between layers l and $l+1$ is located at z_l. We then introduce two layer-specific matrices $\hat{N}_l$ and $\hat{F}_l(z)$:

$$\hat{N}_l=\begin{bmatrix}1 & 1 & 1 & 1\\ \frac{\epsilon_{l,\perp}}{\tilde{k}_{z_{l,1}}} & \frac{\epsilon_{l,\perp}}{\tilde{k}_{z_{l,2}}} & -\frac{\epsilon_{l,\perp}}{\tilde{k}_{z_{l,1}}} & -\frac{\epsilon_{l,\perp}}{\tilde{k}_{z_{l,2}}}\\ \alpha_l(\tilde{k}^2_{z_{l,1}}-\epsilon_{l,\perp}) & \alpha_l(\tilde{k}^2_{z_{l,2}}-\epsilon_{l,\perp}) & \alpha_l(\tilde{k}^2_{z_{l,1}}-\epsilon_{l,\perp}) & \alpha_l(\tilde{k}^2_{z_{l,2}}-\epsilon_{l,\perp})\\ \frac{\tilde{k}^2_{z_{l,1}}-\epsilon_{l,\perp}}{\tilde{k}_{z_{l,1}}} & \frac{\tilde{k}^2_{z_{l,2}}-\epsilon_{l,\perp}}{\tilde{k}_{z_{l,2}}} & -\frac{\tilde{k}^2_{z_{l,1}}-\epsilon_{l,\perp}}{\tilde{k}_{z_{l,1}}} & -\frac{\tilde{k}^2_{z_{l,2}}-\epsilon_{l,\perp}}{\tilde{k}_{z_{l,2}}}\end{bmatrix},$$

$$\hat{F}_l(z)=\begin{bmatrix}\exp(ik_{z_{l,1}}z) & 0 & 0 & 0\\ 0 & \exp(ik_{z_{l,2}}z) & 0 & 0\\ 0 & 0 & \exp(-ik_{z_{l,1}}z) & 0\\ 0 & 0 & 0 & \exp(-ik_{z_{l,2}}z)\end{bmatrix}, \tag{S3}$$

with the columns of the first matrix representing the field distributions of individual modes in the layer, the rows representing the field components of these modes (E_x, B_y, $\alpha_l \partial E_z/\partial z$, and E_z, respectively), and the diagonal elements of the second matrix representing the phase factors of the modes.

With the notations above, the boundary conditions between nonlocal layers l and $l+1$ reduce to:

$$\hat{N}_l \hat{F}_l(z_l)\vec{c}_l = \hat{N}_{l+1}\hat{F}_{l+1}(z_l)\vec{c}_{l+1} \tag{S4}$$

where we combined the amplitudes of the (four) waves propagating in the layer l into a column vector $\vec{c}_l = \{c^+_{l;1}, c^+_{l;2}, c^-_{l;1}, c^-_{l;2}\}$.

When the layer stack contains only nonlocal components, Eq.(S4) can be used to introduce the interface transfer matrix, $\hat{T}_l = \hat{F}_{l+1}(z_l)^{-1}\hat{N}^{-1}_{l+1}\hat{N}_l\hat{F}_l(z_l)$ that relates the amplitudes of the plane waves in the neighboring layers: $\vec{c}_{l+1} = \hat{T}_l\vec{c}_l$.

This relationship can be used to derive the dispersion of the modes in periodically stratified materials. For the bi-layer periodic layered composite with layer thickness d, the overall transfer matrix of one period becomes:

$$\hat{T}_\Delta = \hat{F}_1(d)\hat{N}_1^{-1}\hat{N}_2\hat{F}_2(d)\hat{N}_2^{-1}\hat{N}_1, \tag{S5}$$

with dispersion of the Bloch modes given by Eq.(9) of the main manuscript.

For nonlocal composites with a finite number of layers, transfer matrices can be used to relate the amplitudes of waves in the first layer to the amplitudes in the last layer. The resulting relationship can then be used to solve for the amplitudes of the overall reflected and transmitted waves in therms of the amplitudes of incident waves, followed by calculation of the amplitudes of the waves throughout the layered composite.

The TMM describing the properties of the layered stacks comprising exclusively local components[S2,S3] can be recovered by using 2×2 material matrices,

$$\hat{N}_l = \begin{bmatrix} 1 & 1 \\ \frac{\epsilon_{l,\perp}}{\tilde{k}_{z_l}} & -\frac{\epsilon_{l,\perp}}{\tilde{k}_{z_l}} \end{bmatrix}, \hat{F}_l(z) = \begin{bmatrix} e^{ik_{z_l}z} & 0 \\ 0 & e^{-ik_{z_l}z} \end{bmatrix}, \tag{S6}$$

and using two-component column vector of mode amplitudes $\vec{c}_l = \{c^+_l, c^-_l\}$.

The case of the multi-layer stacks containing both local and nonlocal components where the number of modes changes across the composite is most conveniently described using scattering- (as opposed to transfer-) matrix formalism.[S3]

Within the scattering-matrix framework, the boundary conditions are written in the form given by Eq.(S4), with the $\hat{F}_l(z)\vec{c}_l$ being either two- or four-component vectors (for local and non-local layers respectively), while the material matrices $\hat{N}_l$ are selected according to the following rules that reflect the different number of ABCs required at different interfaces:

- for nonlocal-nonlocal material interfaces $\hat{N}_l$ is given by Eq.(S3),
- for local-local layer interfaces $\hat{N}_l$ is given by Eq.(S6),
- for local component of the local-nonlocal interface, $\hat{N}_l = \begin{bmatrix} 1 & 1 \\ \frac{\epsilon_{l,\perp}}{\tilde{k}_{z_l}} & -\frac{\epsilon_{l,\perp}}{\tilde{k}_{z_l}} \\ 0 & 0 \end{bmatrix}$,
- lastly, for nonlocal component of the local-nonlocal interface,
$$\hat{N}_l = \begin{bmatrix} 1 & 1 & 1 & 1 \\ \frac{\epsilon_{l,\perp}}{\tilde{k}_{z_{l,1}}} & \frac{\epsilon_{l,\perp}}{\tilde{k}_{z_{l,2}}} & -\frac{\epsilon_{l,\perp}}{\tilde{k}_{z_{l,1}}} & -\frac{\epsilon_{l,\perp}}{\tilde{k}_{z_{l,2}}} \\ \alpha_l\left(\tilde{k}^2_{z_{l,1}} - \epsilon_{l,\perp}\right) & \alpha_l\left((\tilde{k}^2_{z_{l,2}} - \epsilon_{l,\perp}\right) & \alpha_l\left((\tilde{k}^2_{z_{l,1}} - \epsilon_{l,\perp}\right) & \alpha_l\left((\tilde{k}^2_{z_{l,2}} - \epsilon_{l,\perp}\right) \end{bmatrix}.$$

The resulting boundary conditions are then used to solve for the amplitudes of the outgoing modes (c^-_l, c^+_{l+1}) in terms of the amplitudes of the incoming modes (c^+_l, c^-_{l+1}). On the implementation level, it is convenient to separate the resulting relationship into "transmission" and "reflection" matrices that relate c^+_{l+1} and c^-_l amplitudes to the amplitude of the incident fields, respectively, and to iteratively calculate the amplitudes of the fields, starting at the last interface of the stack (where $c^-_{l+1} = 0$), and working towards the first interface of the stack. A sample code providing such an implementation can be found in.[S4]

Additional Information on Nonlocality-Induced Optical Transitions

Here we provide additional information on the behavior of the optical modes supported by the nonlocal multilayered composites as the composite undergoes transition from local to nonlocal regimes. We specifically focus on the transition between local effective medium theory, primordial metamaterials, and homogenizable nonlocality.

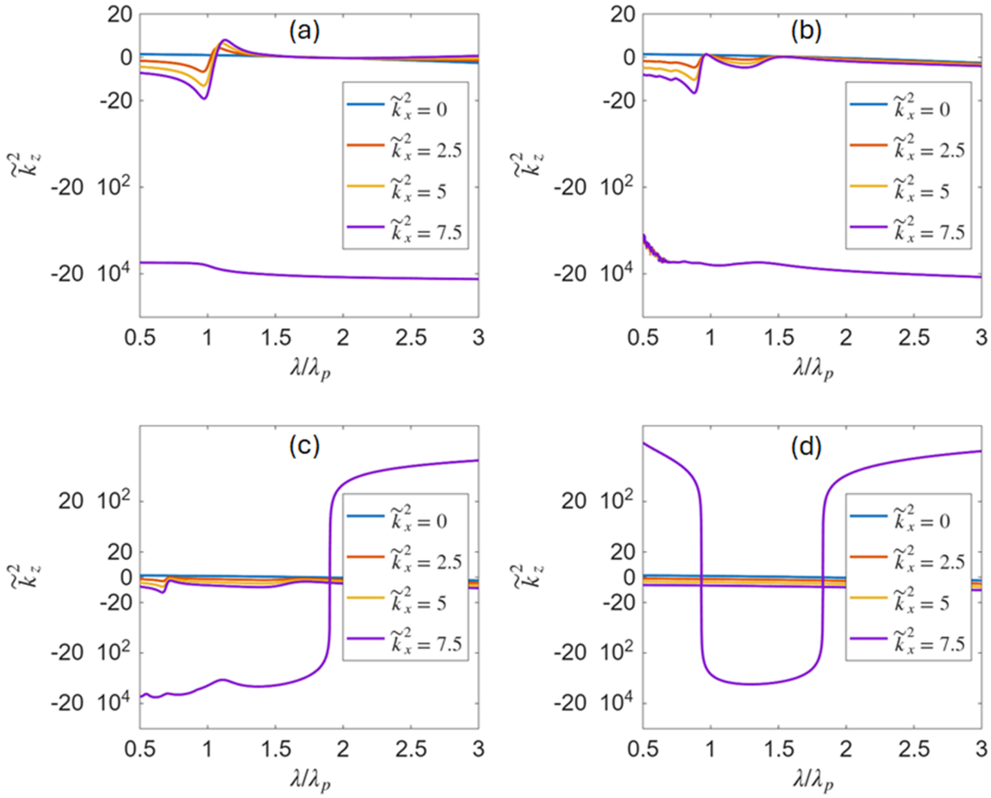

Figure S1: Dispersion of the modes in nonlocal plasmonic/dielectric composites as a function of wavelength, for fixed in-plane wavenumbers (legends). Individual panels represent different composites with layer thickness $d = 0.05\lambda_p$ (a), $d = 0.003\lambda_p$ (b), $d = 10^{-3}\lambda_p$ (c), and $d = 10^{-4}\lambda_p$ (d), calculated using nonlocal transfer matrix method

Fig.S1 illustrates the dispersion of the modes for different values of the in-plane wavenumber. Elliptic-to-hyperbolic transition, that can be described by the local effective medium theory, is clearly visible in Fig.S1(a). As the size of the layer is reduced, inherent nonlocality yields to the spliting of the resonance associated with this transition, and to changes in the behavior of nonlocal mode.

Fig.S2 further illustrates the properties of the two modes supported by the multilayered structure as the composite transitions from local-dominated to nonlocal-dominated regimes. In the local regime, the additional wave is highly lossy. As the size of the components is reduced, the real part of the propagation constant of the nonlocality-dominated mode grows and becomes comparable with its imaginary part. This transition, that represents transition of the additional mode from decaying to oscillating on the scale of the period, corresponds to the onset of the primordial metamaterial regime. Eventually, the real part of propagation constant of the nolocality-dominated mode dominates its propagation.

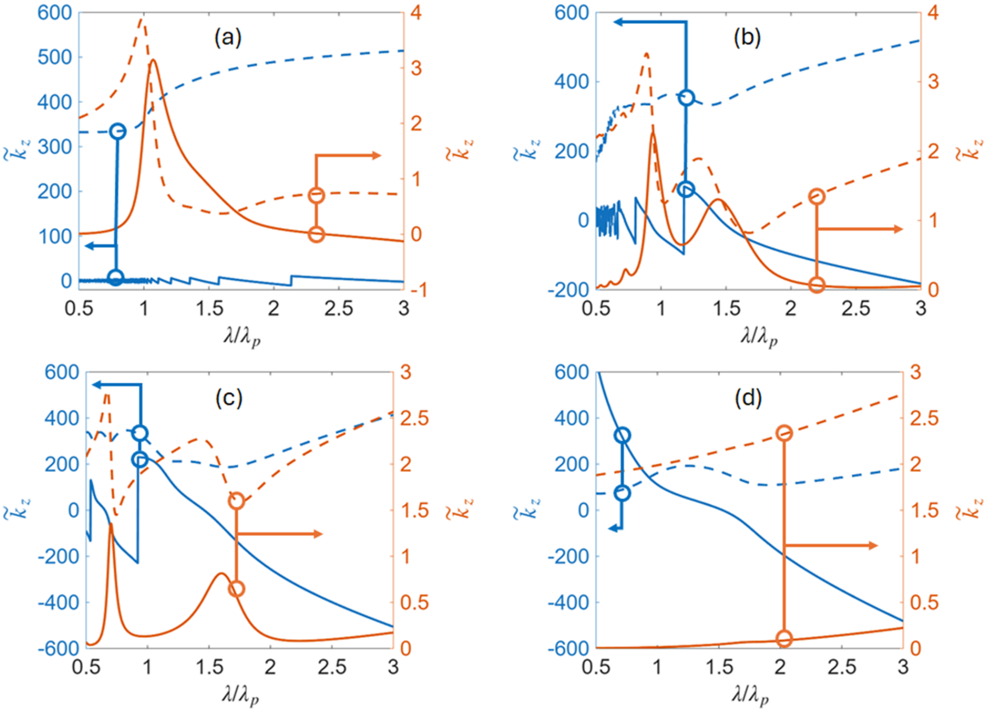

Figure S2: Dispersion of the modes in nonlocal plasmonic/dielectric composites as a function of wavelength, for fixed in-plane wavenumber $\tilde{k}_x^2 = 5$. Individual panels represent different composites with layer thickness $d = 0.05\lambda_p$ (a), $d = 0.003\lambda_p$ (b), $d = 10^{-3}\lambda_p$ (c), and $d = 10^{-4}\lambda_p$ (d), calculated using nonlocal transfer matrix method; left and right axes represent evolution of the two modes that in the weakly-nonlocal homogeneous materials map to additional and main waves, respectively; solid and dashed lines represent real and imaginary part of propagation constant

References

(S1) Landau, L.; Lifshitz, E.; Pitaevskii, L. Electrodynamics of Continuous Media; Permagon Press, Oxford, 1984.

(S2) Yeh, P.; Yariv, A.; Hong, C.-S. J. Opt. Soc. Am. **1977**, 67, 423–438.

(S3) Langevin, D.; Bennet, P.; Khaireh-Walieh, A.; Wiecha, P.; Teytaud, O.; Moreau, A. J. Opt. Soc. Am. B **2024**, 41, A67–A78.

(S4) https://github.com/viktor-podolskiy/primordial_metamaterials.
